# The Metabolic Score for Insulin Resistance (METS-IR) as a Predictor of Incident Ischemic Heart Disease: A Longitudinal Study among Korean without Diabetes

**DOI:** 10.3390/jpm11080742

**Published:** 2021-07-28

**Authors:** Jihyun Yoon, Donghyuk Jung, Yongjae Lee, Byoungjin Park

**Affiliations:** 1 Department of Family Medicine, Yongin Severance Hospital, 363 Dongbaekjukjeondae-ro, Yongin-si 16995, Korea; ghyunyoon@yuhs.ac (J.Y.); balsan2@yuhs.ac (D.J.); 2 Department of Family Medicine, Gangnam Severance Hospital, 211 Eonju-ro, Seoul 06273, Korea; ukyjhome@yuhs.ac

**Keywords:** metabolic score for insulin resistance, cardiometabolic risk, longitudinal study, ischemic heart disease

## Abstract

The metabolic score for insulin resistance (METS-IR) is a novel noninsulin-based marker for assessing the risk of insulin resistance and cardiometabolic risk. However, whether METS-IR is associated with incident ischemic heart disease (IHD) risk is not well known. Therefore, we aimed to investigate the longitudinal effect of METS-IR on incident IHD risk in a large cohort of Korean adults without diabetes. Data were assessed from 17,943 participants without diabetes from the Health Risk Assessment Study (HERAS) and Korea Health Insurance Review and Assessment (HIRA) data. The participants were divided into four groups according to METS-IR index quartiles: (ln ((2 × fasting plasma glucose) + triglyceride) × body mass index)/(ln (HDL-cholesterol)). We prospectively assessed hazard ratios (HRs) with 95% confidence intervals (CIs) for IHD using multivariate Cox proportional hazard regression models over a 50-month period. During the follow-up period, 332 participants (1.9%) developed IHD. HRs of IHD for METS-IR quartiles 1–4 were 1.00, were 1.62 (95% CI 1.04–2.53), 1.87 (95% CI 1.20–2.91), and 2.11 (95% CI 1.35–3.30), respectively, after adjusting for potential confounding variables. A higher METS-IR precedes future IHD among Koreans without diabetes. Moreover, compared with metabolic syndrome, METS-IR had a better predictive value for IHD.

## 1. Introduction

Cardiovascular diseases (CVDs) are the leading cause of death worldwide in 2019, and the majority of deaths from CVD are caused by ischemic heart disease (IHD), with most deaths occurring between the ages of 30 and 70 [1,2]. IHD is a major cause of rising medical expenses, and the early onset of IHD in the aging population is important because it is one of the factors that lowers the quality of life and increases the burden of social medical expenses [3].

Insulin resistance is defined as an impaired biological response to insulin actions in the insulin-responsive tissues and is considered key to the mechanism of metabolic syndrome [4]. The prevalence of insulin resistance has increased globally, and it is known to be from 15.5 to 46.5% of adults [5]. Previous studies have suggested that insulin resistance is significantly related to the development and progression of coronary atherosclerosis and adverse plaque characteristics and is a major risk factor for cardiovascular diseases via pathophysiological mechanisms [4]. Insulin resistance is also the common pathophysiology of prehypertension and prediabetes [6]. Moreover, some studies have found that nondiabetic individuals with IHD tend to exhibit poorer prognosis than diabetic patients without IHD [7,8]. Thus, early detection of insulin resistance in the early stages of IHD is necessary in, for example, non-diabetes patient with metabolic risks and with a high risk of IHD, prevent other diseases and reduce the socioeconomic burden for IHD. 

Recently, the metabolic score for insulin resistance (METS-IR), a higher concordance with the hyperinsulinemic-euglycemic clamp, has been developed, and it has been reported that METS-IR is strongly associated with hypertension and predictive abilities for type 2 diabetes [9,10]. However, to our knowledge, information is limited to the longitudinal association between METS-IR and incident IHD. Therefore, we prospectively investigated the association between METS-IR and IHD incidence in a large-scale, community-dwelling Korean population without diabetes using the Health Risk Assessment Study (HERAS) and Korea Health Insurance Review and Assessment Service (HIRA) database.

## 2. Materials and Methods

### 2.1. Study Population

**Figure 1:**
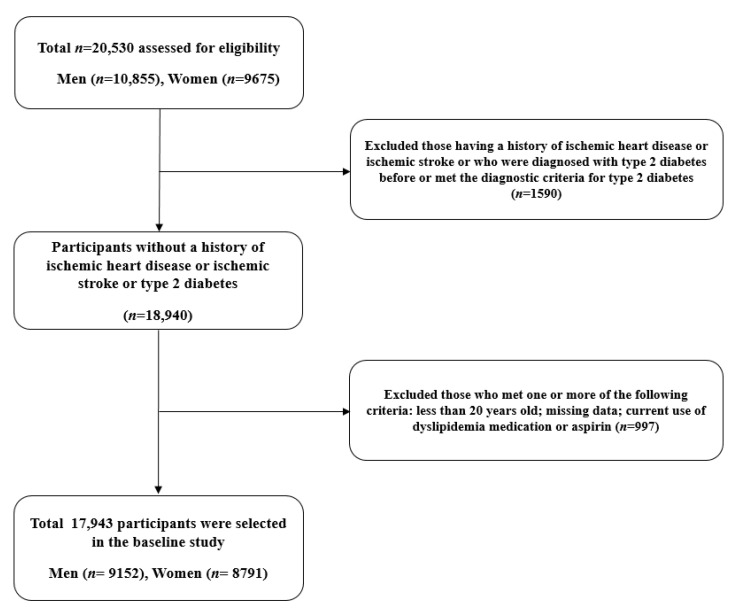
Flowchart for the selection of study participants.

This cohort study was derived from the HERAS-HIRA datasets, aiming to explore surrogate markers for IHD among Korean without diabetes [11]. The cohort consisted of 20,530 subjects who visited the Health Promotion Center at the Yonsei University Gangnam Severance Hospital for health examinations between November 2006 and June 2010. We excluded 1590 participants who had previously been diagnosed with IHD or ischemic stroke, had a previous diagnosis of type 2 diabetes, or a fasting plasma glucose (FPG) level ≥ 126 mg/dL [12]. In addition, patients who met at least one of the following criteria were excluded: aged < 20 years and with current use of dyslipidemia medication or aspirin (*n* = 997). Consequently, 17,943 individuals (9152 men and 8791 women) were included in the final analysis (Figure 1). 

Each participant completed a questionnaire describing their lifestyle and medical history. Smoking status was classified as never smoker, ex-smoker, or current smoker. A regular alcohol drinker was defined as a person who consumed more than 140 g of alcohol per week. Regular exercise was defined as moderate physical activity three or more times per week. Bodyweight and height were measured in light indoor clothing and no shoes to the nearest 0.1 kg and 0.1 cm. Body mass index (BMI) was calculated as weight divided by height squared (kg/m^2^). Systolic blood pressure (SBP) and diastolic blood pressure (DBP) were measured in the sitting position after 10 min of rest using a standard mercury sphygmomanometer (Baumanometer, W.A. Baum Co Inc., Copiague, NY, USA). The mean arterial pressure was calculated from the SBP and DBP. Hypertension was defined as an SBP ≥ 140 mmHg, DBP ≥ 90 mmHg, or current hypertension medication use [13]. Impaired fasting glucose was defined as FPG levels between 100 mg/dL and 125 mg/dL [14]. Metabolic syndrome was defined as the presence of ≥3 of the following risk factors: obesity with BMI ≥ 25.0 kg/m^2^, elevated SBP ≥ 130 mmHg, elevated DBP ≥ 85 mmHg, or using an antihypertensive medication; high FPG levels ≥ 100 mg/dL or using diabetes medication; triglyceride (TG) levels ≥ 150 mg/dL; and high-density lipoprotein cholesterol (HDL-C) < 40 mg/dL and < 50 mg/dL for men and women, respectively [15]. An estimated glomerular filtration rate (eGFR) was calculated as 186.3 × (serum creatinine − 1.154) × (age − 0.203) × 0.742 (if female) [16]. METS-IR was calculated as (ln ((2 × FPG) + TG) × BMI)/(ln (HDL-C)) [9].

### 2.2. Study Outcomes

The primary outcome was IHD, which consisted of angina pectoris (ICD-10 code I20) or acute myocardial infarction (ICD-10 code I21) that occurred after enrollment into the study. To define baseline and study outcomes, we linked a personal 13-digit identification number assigned to each participant by the HIRA between 1 November 2006 and 31 December 2010.

### 2.3. Statistical Analysis

METS-IR values were categorized into quartiles as follows: Q1 (≤28.9), Q2 (29.0–33.2), Q3 (33.3–37.9), and Q4 (≥38.0). All data are presented as mean ± standard deviation or percentage. We have used box plots and the Kolmogorov–Smirnov test to evaluate the distribution of the variables. According to the METS-IR quartiles, the baseline characteristics of the study population were compared using an analysis of variance (ANOVA) model for continuous variables and Pearson’s chi-squared test for categorical variables. Kaplan–Meier curves were used to assess the cumulative incidence of IHD. The log-rank test was used to determine whether the distribution of cumulative IHD incidence differed among the groups. In multivariate analysis, after setting the lowest METS-IR value quartile as a reference group, hazard ratios (HRs) and 95% confidence intervals (CIs) for incident IHD were calculated using the Cox proportional hazards regression model after adjusting for potential confounding variables. All analyses were performed using SAS version 9.4 software (SAS Institute Inc., Cary, NC, USA). All statistical tests were two-sided, and statistical significance was set at *p* < 0.05.

## 3. Results

**Figure 2:**
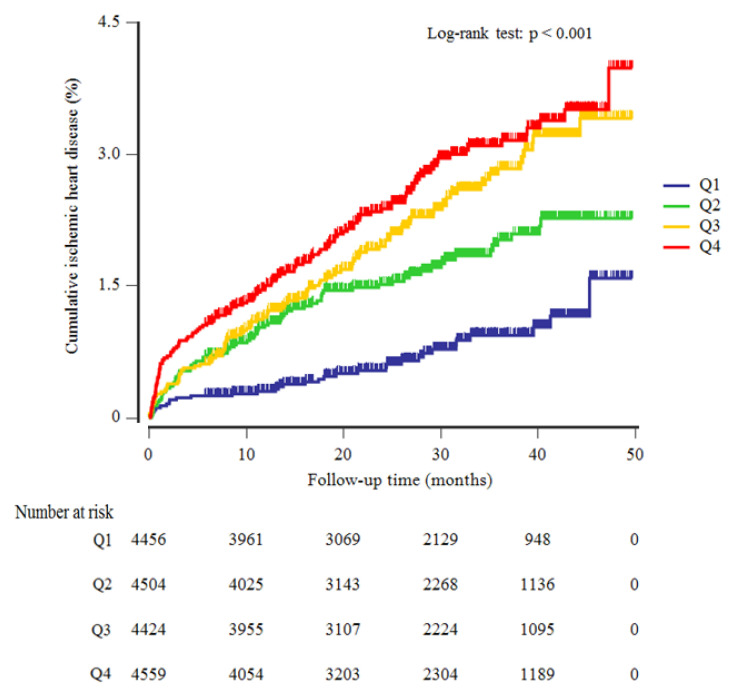
Kaplan–Meier plots indicating the cumulative ischemic heart disease.

Table 1 shows the baseline characteristics of the study population (*n* = 17,943; 9152 men and 8791 women) according to the METS-IR quartiles. The mean age and BMI of the study population were 44.7 ± 10.5 years and 23.3 ± 3.1 kg/m^2^, respectively. The mean FPG concentration was 91.1 ± 9.8 mg/dL, the mean triglycerides level was 122 ± 83 mg/dL, and the mean METS-IR index value was 33.8 ± 6.5. The prevalence of impaired fasting glucose and metabolic syndrome was 17.2% and 11.5%, respectively. Mean BMI, mean arterial pressure, total cholesterol, and high-sensitivity C-reactive protein (hsCRP) values were highest, and mean HDL-C levels and eGFR were lowest in the highest METS-IR index quartile group. The greatest proportion of current smokers and alcohol drinkers were members of the fourth METS-IR index quartile. The higher METS-IR index groups had a significantly elevated cumulative incidence of IHD over a 50-month period that followed the baseline survey (log-rank test, *p* < 0.001) (Figure 2). 

Table 2 shows the results of the multivariate Cox proportional hazards regression analysis for the prediction of IHD according to the METS-IR index quartile. A total of 332 individuals (1.9%, 332/17,943) developed IHD during the study period. The incidence rate (per 1000 person-years) of IHD increased proportionally as the METS-IR index quartile increased. Compared with the first METS-IR index quartile, the HRs of incident IHD for the second, third, and fourth quartiles increased in a dose-dependent manner. The HRs of incident IHD were 1.62 (95% CI 1.04–2.53), 1.87 (95% CI 1.20–2.91), and 2.11 (95% CI 1.35–3.30) for the second, third, and fourth METS-IR index quartiles, respectively, after adjusting for age, sex, smoking status, alcohol intake, physical activity, mean arterial blood pressure, total cholesterol, hsCRP, eGFR, and hypertension medication.

Using a pairwise comparison of receiver operating characteristic (ROC) analyses of incident IHD, the areas under the ROC curves (AUC) of METS-IR data were significantly higher than those of metabolic syndrome (*p* < 0.001), whereas the number of metabolic syndrome was not significantly different. The estimated optimal cut-off values to predict IHD were determined using Youden’s index, with results varying between 0.104 and 0.202. The cut-off value of 31.1, with 81.9 % sensitivity and 38.3 % specificity for METS-IR, seems to be a surrogate marker with a useful screening performance in our study (Table 3). 

## 4. Discussion

Among a community-based population of Korean adults without diabetes, we found that elevated METS-IR was positively and independently associated with IHD incidence in this longitudinal cohort study that included a 50-month follow-up. We also found that METS-IR outperformed the prediction for IHD compared to metabolic syndrome. 

Insulin resistance is described as a low response to insulin action in adipose tissue, skeletal muscles, and liver. In the early stage of insulin resistance, only compensatory hyperinsulinemia appears, and then, in the late stage, insulin resistance can cause the development of dyslipidemia, hypertension, CVDs, etc. [4]. According to pathophysiological mechanisms, insulin is known as the headstream of metabolic syndrome [4]. Insulin resistance is involved in atherosclerosis, and hyperglycemia plays an important role in the early stages of atherosclerosis, which is the main risk factor for developing IHD [17]. Previous studies also revealed that insulin resistance is associated with an increased risk of CVD in nondiabetic patients [18,19]. Thus, early detection of insulin resistance in adults at risk for future IHD is important for prevention and slowing the progression of IHD. HOMA-IR is the most widely used method to evaluate the degree of insulin resistance [20]. However, it is likely to cause bias depending on the use of insulin assay, including calibration setup in the kit and conversions between units [21,22]. Recently, METS-IR, a non-insulin-based insulin resistance, has been reported to have strong predictive abilities for CVD risk [9,10,23,24]. To date, there has been no research on the correlation between METS-IR and IHD.

Metabolic syndrome is said to consist of a cluster of heart disease risk factors, including low HDL-C, high triglyceride, impaired carbohydrate metabolism, central obesity, and high blood pressure [25]. An important feature of metabolic syndrome is insulin resistance, characterized in nondiabetics by increased levels of serum insulin, and it has been suggested that insulin itself is atherogenic [26]. Many epidemiological studies have indicated that metabolic syndrome is associated with IHD and used to predict the risk of IHD in the clinical field [27,28]. Our results are consistent with the findings of previous prospective studies showing that metabolic syndrome was associated with an increased incidence of IHD or CVDs [29,30]. However, the findings of our study showed that the METS-IR had higher predictive power than MetS as a dichotomous classification for IHD. Some possible explanations for this observed association deserve consideration. First, the diagnostic criteria for metabolic syndrome are inconsistent across countries. For example, some studies reported that metabolic syndrome based on Japanese criteria had a weak association with the risk of IHD and predicted IHD less effectively because of the difference in the cutoff values of waist circumference of Japanese metabolic syndrome diagnosis criteria [28]. Second, recent studies have shown that the prognostic role of metabolic syndrome does not increase more than the sum of its components [29,31]. Metabolic syndrome not only increases cardiovascular risk, but also each of its components is associated with an increased risk of CVD [30,32,33,34]. Some studies found that an increased FPG level is a less competent indicator of cardiovascular outcomes [35]. Moreover, the role of BMI in CVDs remains debatable because different studies have presented conflicting results [36,37]. However, studies have demonstrated that triglyceride, HDL-C, glucose intolerance, and insulin levels expectedly correlate best with insulin resistance [38]. It was reported in Korea that obesity is strongly associated with insulin resistance, and a combination of the triglyceride glucose (TyG) index and BMI was superior to other modified TyG indices for predicting insulin resistance in adults [39]. Previous studies have reported that the TyG index may be a useful predictive marker of CVD [11,40]. In addition, triglycerides and HDL-C have each been found to be more predictive of CVD than total cholesterol in the Asia Pacific region [41]. Thus, METS-IR may be regarded as a more favorable predictor of IHD than metabolic syndrome because the combination of triglyceride, BMI, FPG, and HDL-C may lead to a better explanation of the cardiometabolic risk for CVD outcome. Third, some studies have suggested that the risk of cardiovascular disease increases with an increase in the number of metabolic syndrome components [42,43]. Previous studies have suggested that the incidence of coronary heart disease and incident CVD risk shows a progressive increase from one to five metabolic syndrome components [44,45]. Some studies found that the risk of developing CVD increased significantly with increasing number of metabolic syndrome components, and this trend persisted even after adjusting for sex, drinking status, and family history of hypertension, diabetes, and CVD; participants with ≥3 metabolic syndrome components were at three times a higher risk of developing CVD than those without any components [42,43]. We also found that the number of components of metabolic syndrome was more highly predictive of IHD than metabolic syndrome as a dichotomous classification among individuals without diabetes. Thus, consideration of the number of risk components of metabolic syndrome may be more informative than metabolic syndrome as a dichotomous classification when determining the risk of IHD. 

A significant strength of the work was that we conducted a cohort study using many Korean individuals linked to HIRA data from the universal coverage system in Korea. However, the HERAS-HIRA dataset assessed only newly developed IHD and not coronary angioplasty, myocardial resuscitation, or sudden death. Additionally, some individuals with diabetes may have been included in the study population because hemoglobin A1c and 2-h postprandial glucose tests were not available at the baseline.

## 5. Conclusions

An elevated METS-IR predicts future IHD among community-dwelling Koreans without diabetes and is superior to metabolic syndrome as a helpful predictive indicator of IHD. Accordingly, higher METS-IR may be a useful additional measure to assess cardiometabolic risk in nondiabetic adults at the preclinical stage. 

## Figures and Tables

**Table 1:** Baseline characteristics of the study population according to the METS-IR quartiles.

	METS-IR Quartiles					
	Q1 *n* = 4456	Q2 *n* = 4504	Q3 *n* = 4424	Q4 *n* = 4559	*p* Value ^1^	Post Hoc ^2^
METS-IR	≤28.9	29.0–33.2	33.3–37.9	≥38.0		
Age (years)	40.7 ± 10.4	45.4 ± 10.3	46.7 ± 10.1	46.0 ± 10.1	<0.001	a,b,c,d,e,f
Male sex (%)	20.5	42.5	64.0	76.6	<0.001	-
Body mass index (kg/m^2^)	19.8 ± 1.4	22.3 ± 1.3	24.1 ± 1.4	26.8 ± 2.3	<0.001	a,b,c,d,e,f
Systolic blood pressure (mmHg)	113 ± 13	119 ± 14	124 ± 14	130 ± 14	<0.001	a,b,c,d,e,f
Diastolic blood pressure (mmHg)	70 ± 9	74 ± 9	77 ± 9	81 ± 9	<0.001	a,b,c,d,e,f
Mean arterial pressure (mmHg)	84 ± 10	89 ± 10	93 ± 10	97 ± 10	<0.001	a,b,c,d,e,f
Fasting plasma glucose (mg/dL)	86.3 ± 8.4	89.9 ± 8.5	92.4 ± 9.2	95.6 ± 10.3	<0.001	a,b,c,d,e,f
Total cholesterol (mg/dL)	180 ± 31	186 ± 32	191 ± 33	197 ± 34	<0.001	a,b,c,e,f
Triglyceride (mg/dL)	73 ± 26	95 ± 39	125 ± 57	193 ± 117	<0.001	a,b,c,d,e,f
HDL-cholesterol (mg/dL)	65 ± 11	56 ± 10	49 ± 8	43 ± 7	<0.001	a,b,c,d,e,f
C-reactive protein (mg/L)	0.8 ± 2.7	1.2 ± 3.5	1.6 ± 4.6	1.9 ± 4.0	<0.001	a,b,c,d,e,f
eGFR (mL/min/1.73 m^2^)	86.6 ± 14.7	83.9 ± 13.3	82.7 ± 13.0	82.0 ± 12.6	<0.001	a,b,c,d,e
Current smoker (%)	14.6	20.5	26.6	37.4	<0.001	-
Alcohol drinking (%)	35.1	40.5	48.2	51.6	<0.001	-
Regular exercise (%)	26.5	33.6	33.2	27.8	<0.001	-
Hypertension (%)	6.3	13.5	23.3	35.5	<0.001	-
Impaired fasting glucose (%)	5.6	12.0	19.4	31.5	<0.001	-
Metabolic syndrome (%)	0.1	1.5	7.5	36.5	<0.001	-

^1^*p*-values were calculated using one-way ANOVA or Pearson’s chi-square test. ^2^ Post hoc analysis with the Bonferroni method: a, Q1 versus Q2; b, Q1 versus Q3; c, Q1 versus Q4; d, Q2 versus Q3; e, Q2 versus Q4; and f, Q3 versus Q4.

**Table 2:** Hazard ratios and 95% CIx for new-onset IHD according to METS-IR quartiles.

	METS-IR Quartiles				
	Q1	Q2	Q3	Q4	*p* Trend
New cases of ischemic heart disease, *n*	33	76	102	121	
Mean follow-up, years	2.3 ± 1.0	2.4 ± 1.1	2.4 ± 1.1	2.4 ± 1.1	
Person-years of follow-up	10,311	10,646	10,521	10,853	
Incidence rate/1000 person-years	3.2	7.1	9.7	11.1	
Model 1	1.00 (reference)	1.60 (1.06–2.41)	1.91 (1.28–2.86)	2.25 (1.51–3.35)	<0.001
Men	1.00 (reference)	1.55 (0.80–3.04)	2.00 (1.06–3.77)	2.26 (1.21–4.24)	0.031
Women	1.00 (reference)	1.62 (0.95–2.75)	1.71 (0.97–3.00)	2.13 (1.20–3.79)	0.080
Model 2	1.00 (reference)	1.65 (1.06–2.58)	2.00 (1.30–3.01)	2.34 (1.52–3.59)	0.001
Men	1.00 (reference)	1.47 (0.75–2.88)	1.78 (0.94–3.37)	2.14 (1.14–4.03)	0.050
Women	1.00 (reference)	1.73 (0.95–3.15)	2.05 (1.10–3.81)	2.05 (1.06–3.96)	0.111
Model 3	1.00 (reference)	1.63 (1.04–2.54)	1.94 (1.25–3.01)	2.22 (1.43–3.47)	0.004
Men	1.00 (reference)	1.42 (0.72–2.80)	1.70 (0.89–3.25)	2.04 (1.07–3.87)	0.095
Women	1.00 (reference)	1.78 (0.98–3.25)	2.10 (1.12–3.93)	2.11 (1.06–4.20)	0.103
Model 4	1.00 (reference)	1.62 (1.04–2.53)	1.87 (1.20–2.91)	2.11 (1.35–3.30)	0.010
Men	1.00 (reference)	1.39 (0.70–2.73)	1.61 (0.84–3.07)	1.90 (1.00–3.61)	0.169
Women	1.00 (reference)	1.80 (0.99–3.28)	2.07 (1.10–3.88)	2.07 (1.04–4.12)	0.116

Model 1: adjusted for age and sex. Model 2: adjusted for age, sex, smoking status, alcohol intake, and physical activity. Model 3: adjusted for age, sex, smoking status, alcohol intake, physical activity, mean arterial blood pressure, total cholesterol, high-sensitivity C-reactive protein, and eGFR. Model 4: adjusted for age, sex, smoking status, alcohol intake, physical activity, mean arterial blood pressure, total cholesterol, high-sensitivity C-reactive protein, eGFR, and hypertension medication.

**Table 3:** METS-IR versus MetS and the number of MetS components for predicting IHD.

	Pairwise Comparison of AUC					
	Difference		95% CI		*p* Value	
METS-IR vs. MetS	0.069		0.04 to 0.9		<0.001	
METS-IR vs. N of MetS components	0.004		−0.02 to 0.03		0.733	
N of MetS components vs. MetS	0.064		0.04 to 0.09		<0.001	
	**Prediction for Ischemic Heart Disease**					
	**Sensitivity (%)**	**Specificity (%)**	**Cutoff Value**	**AUC**	**Youden’s Index**	***p* Value**
METS-IR	81.9	38.3	>31.1	0.620	0.202	<0.001
Men	84.4	25.4	>32.3	0.554	0.097	0.005
Women	71.9	55.5	>30.9	0.657	0.274	<0.001
MetS	22.7	88.7	>0	0.552	0.104	<0.001
N of MetS components	78.6	39.0	>0	0.616	0.176	<0.001

AUC, area under the receiver operating characteristic curve; MetS, metabolic syndrome; N, number.

## Data Availability

The data underlying this article will be shared upon reasonable request from the corresponding author.
